# Human–Wildlife Conflict Mitigation Based on Damage, Distribution, and Activity: A Case Study of Wild Boar in Zhejiang, Eastern China

**DOI:** 10.3390/ani14111639

**Published:** 2024-05-30

**Authors:** Junchen Liu, Shanshan Zhao, Liping Tan, Jianwu Wang, Xiao Song, Shusheng Zhang, Feng Chen, Aichun Xu

**Affiliations:** 1 College of Life Sciences, Yangtze River Delta Institute of Biodiversity Conservation and Utilization, China Jiliang University, Hangzhou 310018, China; 2 Zhejiang Forest Resources Monitoring Center, Hangzhou 310020, China; 3 The Management Center of Wuyanling National Natural Reserve in Zhejiang, Wenzhou 325500, China

**Keywords:** human–wildlife conflict, wild boar (*Sus scrofa*), damage compensation, Eastern China

## Abstract

**Simple Summary:**

Human–wildlife conflict refers to conflicts that arise from the occurrence or behavior of wildlife that poses a threat to humans directly or indirectly. The wild boar, as one of the most widely distributed ungulates in the world, is known to dominate the species in mountain ecosystems in China. Here, we integrated data collected from damage and camera trap surveys to understand the damage status, abundance and density and activity rhythms of wild boar in Zhejiang, Eastern China. The damage distribution, density and activity rhythms provide specific priority management regions and activity intensity peaks for conflict mitigation. We believe that these findings could provide a scientific basis for mitigation management.

**Abstract:**

Human–wildlife conflicts are becoming increasingly common worldwide and are a challenge to biodiversity management. Compared with compensatory management, which often focuses on solving emergency conflicts, mitigation management allows decision-makers to better understand where the damage is distributed, how the species are distributed and when the species conduct their activity. Here, we integrated data collected from 90 districts/counties’ damage surveys and 1271 camera traps to understand the damage status, abundance, density and activity rhythms of wild boar (*Sus scrofa*) in Zhejiang, Eastern China, from January 2019 to August 2023. We found that (1) wild boar–human conflicts were mainly distributed in the northwest and southwest mountainous regions of Zhejiang Province; (2) the total abundance of wild boar was 115,156 ± 24,072 individuals, indicating a growing trend over the past decade and a higher density in the western and southern regions; (3) wild boar exhibited different activity patterns across different damage regions, and the periods around 7:00, 11:00 and 16:00 represented activity peaks for wild boar in seriously damaged regions. The damage distribution, density, distribution and activity rhythms provide specific priority regions and activity intensity peaks for conflict mitigation. We believe that these findings based on the damage, distribution and activity could provide a scientific basis for mitigation management at the county level and enrich the framework of human–wildlife conflict mitigation.

## 1. Introduction

Human–wildlife conflict refers to conflicts that arise from the occurrence or behavior of wildlife that not only poses a threat to humans but also impacts their well-being. Such conflicts have negative impacts on both humans and wildlife [1] and even threaten biodiversity in many ways [2,3]. A great number of human–wildlife conflict reports have appeared in recent years [4]. There are dozens of species related to conflicts, such as Tibetan brown bears (*Ursus arctos*), Asian elephants (*Elephas maximus*), pumas (*Puma concolor*), wild boar (*Sus scrofa*) and others [5,6,7]. Various driving factors could lead to human–wildlife conflicts both directly and indirectly, and they can be divided into three aspects. (1) The implementation of ecological projects, such as natural forest protection, grain for green, nature reserves, and wildlife preservation [8], has led to continuous improvements in the quality of most habitats for wildlife. Consequently, the populations of wildlife have been steadily increasing, and their activities have expanded. (2) Human activities that are associated with the continuous utilization of natural resources have resulted in the fragmentation of wildlife habitats [9,10]. This has led to overlaps in habitats between humans and wildlife, and the fragmentation intensifies in the process of urbanization [11], thereby increasing the frequency with which wildlife enters cities and the conflicts between these two groups. (3) Climate change acts as an amplifier of conflict by exacerbating resource scarcities [12,13], which could cause increasing interactions between humans and wildlife by changing human–wildlife behavior and distributions [14]. In recent years, conflicts between humans and wildlife have become one of the key ecological issues in China [15]. The question of how to effectively protect humans and property while also safeguarding wildlife remains a significant challenge [4,16].

In comparison to compensatory management, which often focuses on addressing emergency conflicts, precautionary management allows decision-makers to better understand wildlife itself, such as where the damage is distributed, how the species are distributed and when the species conduct their activity [17]. Many studies on human–wildlife conflict have focused on the regional conflict status [18,19], mitigation measure development [20,21], the identification and prediction of risk areas [7,22], the cognition and tolerance of residents [23,24] and other areas, including both single aspects and multiple aspects. Moreover, adding a social perspective (identification tolerability and other key factors), public participation and shared governance could provide other perspectives in mitigation management [25]. However, few studies have been conducted that address human–wildlife conflict mitigation by integrating the damage, distribution and activity, which could enrich the human–wildlife conflict mitigation theory and frameworks.

The wild boar, as one of the most widely distributed ungulates in the world, is known to dominate the species in the mountain ecosystems in China [26]. Since the construction of a series of projects, such as the protection and restoration of natural forests and the return of farmland to forests [27], wild boar has been considered as the primary species that comes into conflict with humans [28]. Moreover, the shrinking distribution areas and declining numbers of tigers (*Panthera tigris*), clouded leopards (*Neofelis nebulosa*), wolves (*Canis lupus*) and other species have resulted in reduced hunting pressures faced by wild boar [29]. In addition, because of their omnivorous diets and strong reproductive capabilities [17,19,30], the population of wild boar has rapidly increased in recent years. Consequently, they have become notorious for causing extensive damage to crops, posing risks to human safety and spreading disease in many regions [31,32]. From 2000 to 2023, wild boar was listed among the “Terrestrial Wildlife with Important Ecological, Scientific and Social Value” in China; thus, it is protected by Chinese law and prohibited from being captured or killed. However, the National Forestry and Grassland Administration removed wild boar from the updated list in June 2023 [33]. This decision was intended to provide more space for comprehensive strategies to manage the growing abundance of wild boar and mitigate the associated conflicts. In our study, wild boar refers to purely wild forms and not pig–boar hybrids or feral hogs, as it plays a key role in ecosystems.

The Yangtze River Delta has emerged as one of the three crucial strategic core urban agglomerations along the Yangtze River Economic Belt [34]. Zhejiang Province is situated at the southern end of the Yangtze River Delta, and its economy has developed rapidly. Zhejiang Province, maintaining a forest coverage rate surpassing 60% [35], provides a suitable habitat for wild boar’s survival and reproduction. In recent years, numerous reports have indicated an increasing trend in the frequency, spatial scope and degree of damage caused by wild boar–human conflicts in Zhejiang Province, according to management authorities and local populations [36]. The government is confronted with the urgent task of controlling the population of wild boar and mitigating its impacts [37]. At the same time, most local residents hold the general belief that wild boar is more numerous and harmful than other species. However, we still lack scientific and effective assessments concerning the population of wild boar and the current situation of the conflict between wild boar and humans.

In this study, we aimed at quantifying the damage status, abundance, density and activity rhythms of this conflict species by integrating data collected from damage surveys and camera traps. We took Zhejiang Province of Eastern China as the study area and the wild boar as the study species. We investigated the wild boar’s damage across 90 districts and/or counties and deployed 1271 infrared cameras. By analyzing the data obtained from the infrared camera and damage surveys, we (1) analyzed the wild boar’s damage and drivers in all districts and counties in Zhejiang Province; (2) analyzed the distribution, population density and abundance of wild boar across the province; and (3) explored the rhythms and characteristics of the wild boar’s activity in different damage regions.

## 2. Material and Methods

### 2.1. Study Area

The Yangtze River Economic Belt spans the eastern, central and western regions of China. It holds a significant share of the population and gross domestic product (GDP), exceeding 40% of the country’s total [38]. Within the Belt, the Yangtze River Delta emerges as one of the three crucial strategic core urban agglomerations. Zhejiang Province, as one of the key urban agglomerations, lies on the southern flank of the Yangtze River Delta (118°01′~123°08′ E, 27°01′~31°10′ N). The total land area is 101,800 km^2^ [39]. The landscape of Zhejiang exhibits distinct characteristics across the different regions. The southwest is predominantly mountainous, and the central part is dominated by hills, while the northeast consists of low and flat alluvial plains [40]. Zhejiang Province has a humid monsoon climate. The weather is hot and rainy in summer and mild and rainy in winter [41]. In terms of the topographic distribution, mountains and hills cover 70.4% of the total area, with the main mountain extending in a southwest–northeast direction [42]. There are 11 cities and 90 districts and counties in Zhejiang Province.

### 2.2. Wild Boar Damage Survey

In order to mitigate human–wildlife conflicts, it is important to understand the spatial and temporal distribution and loss caused by human–wildlife conflicts [43] and predict the potential distribution areas of human–wildlife conflicts [22,44]. In the current study, we collaborated with the local forestry management department to conduct a wild boar damage survey across all districts and counties (n = 90) of Zhejiang Province. Based on the preliminary assessment of the damage caused by wild boar, we classified the damage into two categories: (1) physical injuries to local residents and (2) damage to crops (Table 1). The surveys included gathering data on (1) the number of injuries inflicted by wild boar on residents, (2) the extent of crop damage and (3) the amount of economic losses caused to residents in all 90 districts and counties, which included the damage to crops. In our study area, the economic losses were related to the type and degree of damaged crops.

### 2.3. Camera Trap Survey

**Figure 1:**
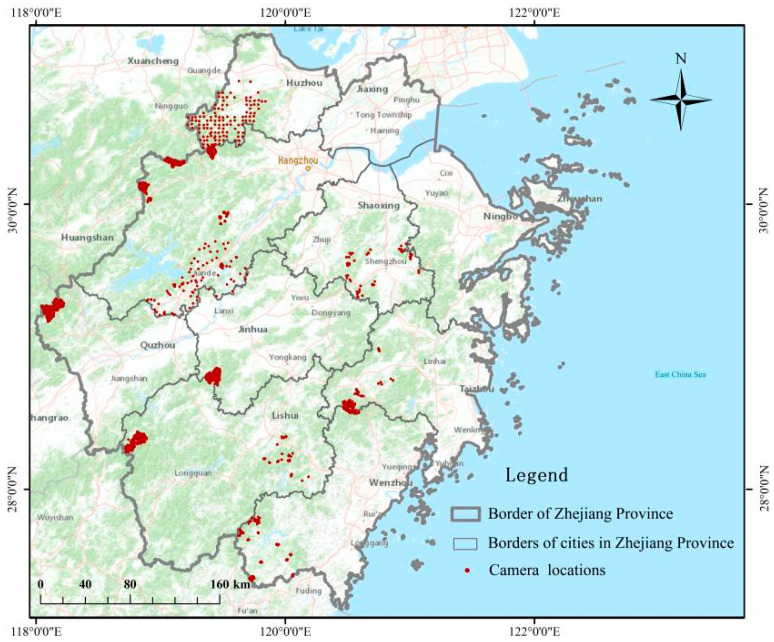
Locations of camera trap stations and sampling areas in Zhejiang Province (due to the map resolution, the points in Figure 1 are fragmented or scattered).

Camera trapping, due to the advantages of accuracy, continuous operation and concealment [45], is widely used to estimate the abundance of species, as well as to investigate their activity rhythms [46,47,48]. The use of cameras might also help in assessing densities, i.e., it may be an important tool to verify data from other sources and, based on temporal trends, allow for actions aimed at minimizing a conflict before it escalates. Camera traps served as the primary means of data collection in the current study. From January 2019 to August 2023, we systematically selected 11 districts/counties (belonging to 8 cities) in Zhejiang Province (Table S1). The total camera survey area was 2761.74 km^2^ (mean ± standard error = 251 ± 144 km^2^). We divided them into fixed cells (1 km × 1 km) based on the GIS raster map. According to the elevation, slope, vegetation and orientation, we used the random sampling method to set infrared cameras (Ltl-6210). A total of 1271 infrared cameras were deployed in our study (Figure 1). In order to minimize the disturbance to animal behavior and prevent excessive animal attraction, we refrained from using lures or baits at the camera stations [49]. The camera settings were configured to capture a continuous sequence of 2 photographs, with a 5 s interval between shots and medium sensitivity. The cameras were fastened to tree trunks approximately 40~80 cm above the ground. We replaced the memory cards and batteries every 6~8 months.

### 2.4. Data Analyses

#### 2.4.1. Wild Boar’s Damage and Its Potential Drivers

We sorted the damage of the 90 districts/counties and calculated the physical injures and damage to crops in each district/county. We scored the levels of injury to residents, damage to crops and economic losses to explore the damage degrees (Table 2). We defined the total damage as the sum of the scores of each damage type. We classified the damaged districts and counties into four categories: severely damaged (25%), moderately severely damaged (25%), generally damaged (25%) and slightly damaged (25%). We used ArcGIS (10.8) to display the damage distribution.

In order to explore the potential factors that drive wild boar’s damage, we analyzed the correlations between four types of damage (the amount of damage to crops, the area of the damage to crops, the amount of economic losses caused by wild boar to residents and the total damage) and four potential factors (the vegetation area, GDP, population and cultivated land area). We obtained the vegetation area and cultivated land of each district/county from the third national land survey of Zhejiang Province [50]. We collected the GDP and population size of each district/county from the Zhejiang Statistical Yearbook [51]. We used the Kolmogorov–Smirnov test to analyze the normality of all variables. We used the Spearman method to conduct a partial correlation analysis because all variables were non-normally distributed. As there were differences in the area of each district/county, we used the area of the district/country as a controlled factor in the partial correlation analysis.

#### 2.4.2. Abundance and Density of Wild Boar

We used the classic random encounter model to estimate the abundance of wild boar [52]. Firstly, we defined records of the same species captured by the same camera as independent when the images were taken at least 30 min apart [53]. The manual identification of images obtained from the infrared cameras was conducted for data analysis. The density of wild boar in our study was estimated based on the random encounter model [54].

$$D=\frac{y}{t}\frac{\pi }{\mathrm{vr}\left(2+\theta \right)}\times g$$

*D* is the density of wild boar (individual/km^2^). *y* is the total number of independent images of wild boar obtained in this research. *t* is the cumulative number of days for which all cameras were working normally. v is the average daily movement speed of wild boar (km/day). After sorting the daily movement distances of wild boar from the studies performed in China, we selected v = 2 km/day in the current study [55,56]. r is the radius of the effective monitoring area of the infrared camera. Combined with the vegetation conditions in the camera locations, we obtained r = 0.005 km. θ is the angle of the infrared camera detection area. The detection angle of the infrared camera used in this study was 0.91 rad (52°). *g* is the wild boar’s group size.

The average density of wild boar across Zhejiang Province was calculated using the weighted average method:

$$\bar{D}=\frac{\sum_{i=1}^{n}\left(D_{i}\times S_{i}\right)}{\sum_{i=1}^{n}S_{i}}$$

$\bar{D}$ represents the average density. $D_{i}$ represents the density of wild boar in the *i* sample survey district/county. $S_{i}$ is the vegetation area of the *i* sample survey district/county. n is the total number of survey districts/counties.

The abundance of wild boar in Zhejiang Province was calculated by summing the abundance of wild boar in each district/county. Following Yu (2021), we multiplied the average density by the vegetation area of each district/county. The vegetation area data of each district/county were extracted from the classified data of the third national land survey of Zhejiang Province [50].

#### 2.4.3. Wild Boar’s Activity Rhythms

In order to explore the relationship between the activity rhythms of wild boar and the different damage levels, we selected two adjacent groups of severely damaged and generally damaged districts and counties and used the kernel density estimation method [57] to analyze the annual daily activity characteristics of wild boar in these two groups of regions. The data used to analyze the wild boar’s activity rhythms covered one year, including the breeding and wintering seasons. The two groups of districts and counties were as follows: in the northern region of Zhejiang Province, Linan District (severely damaged) and Anji County (generally damaged); in the southern region of Zhejiang Province, Suichang County (severely damaged) and Wucheng District (generally damaged). This method considers that each detection of a species is a random sample collected from the continuous distribution of the daily activity rhythms. This daily activity rhythm distribution describes the probability of detecting the species during specific time intervals. The horizontal axis is the time and the vertical axis (density) is the probability of the species being detected at this time point, and the integral value of the area under the curve is 1.

At the same time, we used the coefficient of overlap [58] to calculate the degree of overlap of the daily activity rhythms of wild boar in severely damaged regions and generally severely damaged regions, expressed as the area ratio Δ of the overlapping activity rhythm distribution curves of the two. Δ = 0 indicates complete separation, and Δ = 1 indicates complete overlap.

The kernel density estimation and coefficient of overlap analysis were conducted using the number of independent images as the basis. We used the “overlap” and “activity” packages of R 4.0.3 to conduct the activity analysis.

## 3. Results

### 3.1. Damage Status of Wild Boar and Its Drivers in Zhejiang Province

**Figure 2:**
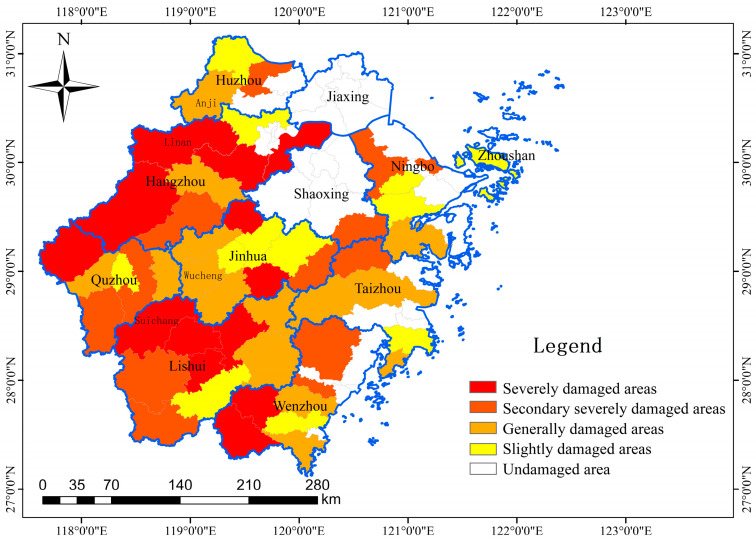
Distribution of wild boar damage in Zhejiang Province.

With the exception of Jiaxing City in Northern Zhejiang Province, damage caused by wild boar occurred in 10 cities. Specifically, wild boar damage occurred in 60% of the districts/counties (54/90) and 27.27% of the cities (3/11, Lishui, Jinhua and Quzhou City) in Zhejiang Province. There were 40 incidents of physical injury, 9593 instances of damage to crops and 33.82 km^2^ of damaged crops caused by wild boar, resulting in economic losses of 74.65 million yuan. We identified three cities (Lishui, Hangzhou and Jinhua) with severe losses based on four indicators: the frequency of damage, the damaged crop area, the amount of damage and the physical injuries (Figure 2). There were no significant correlations between each damage type and each factor (Table S2).

### 3.2. Abundance and Density of Wild Boar in Zhejiang Province

We selected 11 districts and counties in Zhejiang Province, deployed a total of 1271 infrared cameras (116 ± 17 units) and obtained 5226 independent images of wild boar. The density of wild boar in Zhejiang Province is 1.77 ± 0.37 individuals/km^2^ (Table 3).

**Figure 3:**
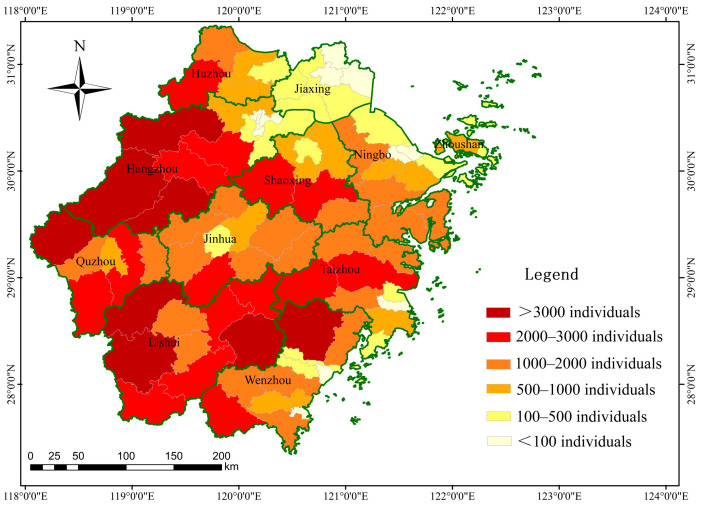
Abundance of wild boar in Zhejiang Province.

The highest density of wild boar appeared in Linan District (3.01 ± 0.57 individuals/km^2^), followed by Xianju County (2.91 ± 0.58 individuals/km^2^) and Wucheng District (2.78 ± 0.34 individuals/km^2^), and the smallest occurred in Tonglu County (0.32 ± 0.10 individuals/km^2^, Table 3, Figure S1). The abundance of wild boar in Zhejiang Province was 115,156 ± 24,072. Among the prefecture-level cities, Lishui City had the largest population (25,199 ± 5268 individuals), followed by Hangzhou City (20,374 ± 4259 individuals) and Wenzhou City (12,927 ± 2702 individuals). We found that more than 5000 individuals occurred in the prefecture-level cities in Zhejiang Province, except Jiaxing and Zhoushan. More than 1000 wild boar individuals were detected in 51.11% (46/90) of the districts and counties. Additionally, 58 districts and counties possessed more than 500 individuals, accounting for 64.44% (Figure 3).

### 3.3. Activity Rhythms of Wild Boar in Zhejiang Province

**Figure 4:**
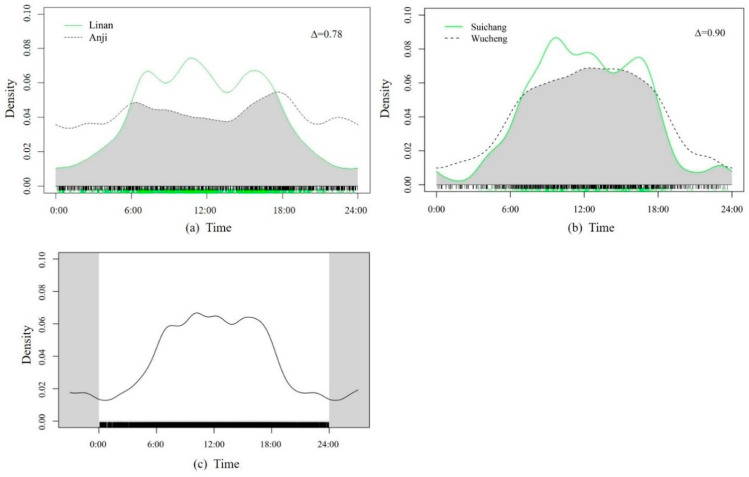
(**a**) Overlap of daily activity rhythms of wild boar in severely damaged (Linan District) and generally damaged (Anji County) regions. (**b**) Overlap of daily activity rhythms of wild boar in severely damaged (Suichang County) and generally damaged (Wucheng District) regions. (**c**) Daily activity rhythms of wild boar in Zhejiang Province.

Our findings revealed that the coefficient of overlap of the daily activity rhythms of wild boar between Suichang County (severely damaged) and Wucheng District (generally damaged) was relatively high (Δ = 0.90), and the coefficient of overlap between Linan District (severely damaged) and Anji County (generally damaged) was relatively low (Δ = 0.78). The activity rhythm of wild boar between Linan District and Suichang County was tripodal and concentrated during the day (6:00–18:00). However, the wild boar in Anji County displayed a bimodal pattern, with activity peaks observed during the morning and dusk, while Wucheng District maintained continuous activity without peaks during the day (Figure 4).

## 4. Discussion

### 4.1. Wild Boar’s Damage and Its Potential Drivers

The assessment of the damage caused by wild boar revealed that all cities in Zhejiang Province experienced such damage, except Jiaxing City. This indicates that the damage caused by wild boar is relatively widespread. The degree of damage inflicted by wild boar is influenced not only by their population size but also by various factors, including food resources, the surrounding vegetation, elevation and so on [23,59,60]. In Zhejiang Province, wild boar predominantly inhabit habitats characterized by a relatively dense canopy, such as deciduous broadleaf forests and mixed coniferous and broadleaf forests found at mid- to high altitudes [61]. These habitats offer favorable conditions with suitable shelter and facilitate the foraging and movement of wild boar [62,63]. No reports of wild boar damage have been published in Jiaxing City. This might be due to the fact that Jiaxing City is located in the Hangzhou–Jiaxing–Huzhou Plain and has a lower altitude with a smaller vegetation area (it accounts for only 0.84% of the entire province).

The crop regions in mountain areas, in comparison with eastern and northern plains, experience serious crop damage due to wild boar (Figure 2). The crops in mountains tend to be sporadically distributed in the mountains, which are close to breeding and foraging areas. Moreover, due to the undeveloped traffic, the crops in the mountains are not seriously influenced by human activity. Our results also show that the top three cities in terms of the area of crops damaged by wild boar are Hangzhou, Lishui and Wenzhou. The cultivated land in these three cities accounts for 32.81% of the entire province. The combined information reveals that crops in mountain areas should be the primary focus for the management and prevention of crop damage caused by wild boar.

Although seriously damaged districts/counties were identified in western and southern Zhejiang Province that possessed a larger vegetation area and lower GDP [50,51], there were no significant correlations between each damage type and each factor. This result might be due to the research scale and the attitudes and cognitions of residents. A regional-scale study in Kaihua of Zhejiang revealed that the crop types, landform, microhabitat, guarding intensity of farmers and other factors could lead to human–wild boar conflicts [64]. The current study was performed at the district/county scale, the findings of which also should be taken into consideration in mitigation management. The attitudes and cognitions of residents might disproportionately cause variations in the damage degree.

### 4.2. Wild Boar’s Abundance and Density

The abundance and density of wild boar have increased over the past two decades in Zhejiang Province. The density of wild boar in Zhejiang Province is 1.77 ± 0.37 individuals/km^2^, higher than the density of 1.38 ± 0.18 individuals/km^2^ surveyed in the area from 2014 to 2016 [65]. The estimated abundance of wild boar in Zhejiang Province is 115,156 ± 24,072 individuals, which is comparable to the abundance in Shaanxi Province, China (128,707 ± 16,718 individuals) [66]. The abundance of wild boar is 3.97 times higher than that in 2000 and 1.15 times higher than that in 2016 (Figure S2). The highest abundance of wild boar is found in Lishui City (25,199 ± 5268 individuals), which is in line with the findings of the “Zhejiang Special Investigation of Wild Boar” [65]. However, it should be noted that the “Zhejiang Special Investigation of Wild Boar” performed in 2016 used the method of transects and infrared cameras [65], which might have had an impact on the estimation of the wild boar’s abundance.

### 4.3. Wild Boar’s Activity Rhythm

An activity rhythm is a biological phenomenon formed by animals to adapt to environmental changes [67]. Wild boar exhibit significant variations in their activity rhythms and possess strong adaptability to external factors [68]. We found that there were significant differences in the daily activity rhythms of wild boar between the severely damaged region (Linan District) and generally damaged region (Anji County) in Northern Zhejiang. The variable activity rhythm hints at the behavioral plasticity of wild boar, which could explain the increasing human–wild boar conflicts in recent years.

The peaks in the wild boar activity patterns between the severely damaged regions (such as Linan District) and generally damaged regions (such as Anji County) are not consistent throughout the year. In generally damaged regions, wild boar exhibit a bimodal activity pattern with peaks at dawn and dusk, and they tend to show higher activity at 5:00–7:00 and 17:00–19:00. In severely damaged regions, wild boar show a three-peak pattern, with higher activity around 7:00, 11:00 and 16:00. Although the elevation, vegetation, predators and other elements are factors influencing the activity rhythms of wildlife [69,70,71], the specific activity patterns of wild boar identified in the different damaged regions in the current study could provide temporal evidence to reduce the encounters between local residents and wild boar.

In our study, wild boar exhibited a concentration of diurnal activity, which is in accordance with observations in Northeastern China [72] and Southwestern China [73]. However, wild boar exhibit nocturnal activity patterns in Central Argentina [17], Central Italy [68] and Northern Germany [74]. This discrepancy may be attributed to the reduced presence of large carnivores in the study area and the implementation of laws that prohibit the use of firearms by residents in China since 1996 [61]. Although there have historically been great numbers of large carnivores in Zhejiang, they have almost disappeared in recent years. No large carnivores (tigers, clouded leopards, wolves) were recorded both during the daytime and the nighttime in our study area. Moreover, the hunting of wild animals is not allowed in China, which alleviates the risk of their death. Consequently, wild boar may not need to evade predators and humans frequently during the daytime [75].

### 4.4. Management Implications

**Figure 5:**
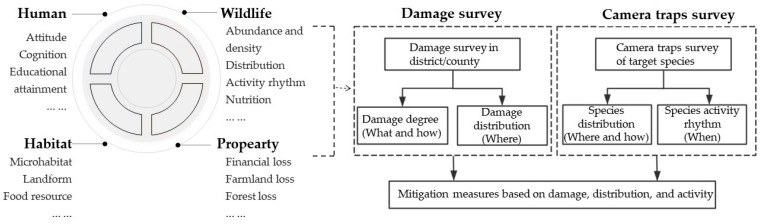
Framework of human–wildlife conflict mitigation. Precautionary management could be conducted based on this framework to determine where and how wild boar’s damage and population are distributed to mitigate conflicts at a district and county level.

Due to the increasing human–wildlife conflicts around the world, it is essential to take several actions to mitigate such conflicts in order to achieve coexistence. Human–wildlife conflicts involve multiple scales, such as humans, habitats, property, animals and so on. In the current study, we integrated data collected from camera traps and field surveys to understand the damage status (degree and distribution), abundance and density (distribution) and activity rhythms (activity intensity/peaks) of the conflict species (Figure 5), which could improve the understanding of the animal and property perspectives.

In our study, at the district and county level, we recommend that the government should pay attention to districts and counties with serious wild boar damage and a high density and enhance the intensity of damage control and formulate macro policies. At the local level, targeted prevention and control efforts should be strengthened in regions experiencing frequent human–wildlife conflicts, such as farmland, orchards and agricultural and forestry areas. Moreover, other practical measures, such as the establishment of electronic fences [76,77], the utilization of sound and light intimidation techniques [20] and interference coupled with legal hunting [78] and planning arrangements, such as the construction of ecological corridors [79] and the adjustment of land use [18], also should be integrated into the current management. Finally, public campaigns targeting residents can be implemented [80]. Meanwhile, the local residents can provide valid support in providing regularly updated data on the fauna, which could create a valuable system that increases the cooperation between the residents and management authorities and their connection with the territory.

### 4.5. Limitations

It is important to note that due to the limitations of the data collection, the survey time of the infrared cameras varied across the different districts and counties included in our study. As the selected districts and counties involved several projects, we attempted to sort the data to shorten the differences in the survey time. This lack of uniformity in the survey time may have an impact on the accuracy of the results. We believe that the damage status (degree and distribution), abundance and density (distribution) and activity rhythms (activity intensity/peaks) based on similar survey times could contribute to mitigating the conflicts.

## 5. Conclusions

We conclude that (1) wild boar–human conflicts are mainly distributed in the northwest and southwest mountainous regions of Zhejiang Province; (2) the total abundance of wild boar is 115,156 ± 24,072 individuals, indicating a growing trend over the past decade and a higher density in the western and southern regions; (3) wild boar exhibit different activity patterns across different damage regions, and the periods around 7:00, 11:00 and 16:00 represent activity peaks for wild boar in seriously damaged regions. The specific damage, abundance/density distribution and activity rhythms (intensity/peaks) of the target species will benefit conflict mitigation. The findings of our study provide a scientific foundation for the control of the wild boar population, damage protection strategies and compensation frameworks at the district and county level.

## Figures and Tables

**Table 1:** The damage research content regarding wild boar in Zhejiang Province.

Category	Content	Description
Physical injuries to local residents	Number of injuries to residents	Number of injuries and casualties caused by wild boar to humans (times)
Damage to crops	Amount of damage to crops	Amount of damage to local crops caused by wild boar (times)
	Area of damage to crops	Area of wild boar damage to local crops (m^2^)
	Amount of economic losses caused by wild boar to residents	Amount of money lost by residents after wild boar caused damage to local crops (CNY), which is related to the type and degree of damaged crops

**Table 2:** Assignment of wild boar damage surveyed across 90 districts/counties in Zhejiang.

Investigation Content	Assignment
Number of injuries to residents	The number of injuries to residents per county: 0 time = 0, 1~2 times = 1, 3~4 times = 2, 5~6 times = 3, 7~8 times = 4, 9~10 times = 5, 11~12 times = 6, more than 13 times = 7
Amount of damage to crops	The amount of damage: 0 time = 0, 1~100 times = 1, 101~200 times = 2, 201~300 times = 3, 301~400 times = 4, 401~500 times = 5, 501~600 times = 6, more than 601 times = 7
Area of damage to crops	The area of damage: 0 Mu = 0, 1~200 Mu = 1, 201~400 Mu = 2, 401~600 Mu = 3, 601~800 Mu = 4, 801~1000 Mu = 5, 1001~1200 Mu = 6, more than 1201 Mu = 7
Amount of economic losses caused by wild boar to residents	The amount of economic losses: 0 million yuan = 0, 1~50 million yuan = 1, 51~100 million yuan = 2, 101~150 million yuan = 3, 151~200 million yuan = 4, 201~250 million yuan = 5, 251~300 million yuan = 6, more than 301 million yuan = 7

Note: 1 Mu ≈ 666.67 m^2^ in China.

**Table 3:** Density of wild boar in each surveyed district and county.

City	District/County	Population Density of Wild Boar (Individuals/km^2^)
Hangzhou	Linan	3.01 ± 0.57
	Jiande	1.25 ± 0.15
	Tonglu	0.32 ± 0.10
Wenzhou	Taishun	0.50 ± 0.11
Huzhou	Anji	1.89 ± 0.41
Shaoxing	Shengzhou	0.68 ± 0.13
Jinhua	Wucheng	2.78 ± 0.34
Taizhou	Xianju	2.91 ± 0.58
Lishui	Qingtian	1.82 ± 0.54
	Suichang	1.47 ± 0.17
Quzhou	Kaihua	2.02 ± 0.73
Average density		1.77 ± 0.37

## Data Availability

The data presented in this study are available upon request from the corresponding author.

## Supplementary Materials

The following supporting information can be downloaded at: https://www.mdpi.com/article/10.3390/ani14111639/s1, Table S1: Survey areas and survey times in Zhejiang of Eastern China. Table S2. Results of partial correlation analysis between damage types and factors in Zhejiang of Eastern China. Figure S1. Density of wild boar in each district and county surveyed in Zhejiang of Eastern China. Figure S2. Changes in wild boar’s abundance in Zhejiang of Eastern China.
