# Supplementary material for: Human–Wildlife Conflict Mitigation Based on Damage, Distribution, and Activity: A Case Study of Wild Boar in Zhejiang, Eastern China
Source: Animals (Basel). 2024 May 30;14(11):1639. doi: 10.3390/ani14111639 (PMC11171170; doi:10.3390/ani14111639)

# Human–Wildlife Conflict Mitigation Based on Damage, Distribution, and Activity: A Case Study of Wild Boar in Zhejiang, Eastern China

**Junchen Liu [1], Shanshan Zhao [1,*], Liping Tan [1], Jianwu Wang [2], Xiao Song [1], Shusheng Zhang [3], Feng Chen [2] and Aichun Xu [1,*]**

[1] College of Life Sciences, Yangtze River Delta Institute of Biodiversity Conservation and Utilization, China Jiliang University, Hangzhou 310018, China
[2] Zhejiang Forest Resources Monitoring Center, Hangzhou 310020, China
[3] The Management Center of Wuyanling National Natural Reserve in Zhejiang, Wenzhou 325500, China
* Correspondence: zhaoss9211@126.com (S.Z.); springlover@cjlu.edu.cn (A.X.)

## Supplementary material Tables

Table S1 Survey areas and survey time in Zhejiang of eastern China.
Table S2 Results of partial correlation analysis between damage types and factors in Zhejiang of eastern China.

**Table S1.** Survey areas and survey time in Zhejiang of eastern China.

| City | District/ County | Survey time | Survey area | The number of cameras | Survey area （km²） |
|---|---|---|---|---|---|
| Hangzhou | Linan | 2021.11-2022.10 | Tianmushan Town, Changhua Town | 118 | 1655.36 |
| | Jiande | 2021.12-2022.02 | Xinanjiang Street, Yangxi Street, Genglou Street, Lianhua Town, Gantan Town, Meicheng Town, Yangcunqiao Town, Xiaya Town, Dayang Town, Sandu Town, Shouchang Town, Hangtou Town, Daciyan Town, Datong Town, Lijia Town, Qintang Town | 102 | 42.74 |
| | Tonglu | 2020.11-2021.10 | Yaolin Forestry Center, Yaolin Town | 95 | 18.34 |
| Wenzhou | Taishun | 2020.05-2021.04 | Luoyang Town, Xiyang Town, sixi Town, Siqian Town | 105 | 148.06 |
| Huzhou | Anji | 2019.01-2019.12 | Meixi Town, Tianzihu Town, Zhangwu Town, Hanggai Town, Xiaofeng Town, Baofu Town, Zhangcun Town, Tianhuangping Town, Xilong Town, Shangshu Town, Shanchuan Town, Dipu Street, Changshuo Street, Lingfeng Street, Xiaoyuan Street. | 139 | 382.28 |
| Shaoxing | Shengzhou | 2022.03-2023.02 | Ganlin Town, Changle Town, Chongren Town, Xiawang Town, Shihuang Town, Gulai Town, Jinting Town, Guimen Town | 66 | 82.89 |
| Jinhua | Wucheng | 2022.09-2023.08 | Tashi Town, Lingsang Town, Shafan Town, Ruoyang Town, Andi Town | 100 | 95.33 |
| Taizhou | Xianju | 2021.12-2022.11 | Danzhu Town, Zhuxi Town, Baita Town, Guangdu Town | 62 | 24.28 |

| City | District/ County | Survey time | Survey area | The number of cameras | Survey area （km²） |
|---|---|---|---|---|---|
| Lishui | Qingtian | 2021.03-2022.02 | Shimen Cave Forestry Center, Zhangcun Town, Zhenwang Town, Haikou Town, Zhenbu Town, Jupu Town, Fushan Town, Lakou Town | 70 | 44.82 |
| | Suichang | 2021.03-2022.02 | Jiulongshan National Nature Reserve | 147 | 127.25 |
| Quzhou | Kaihua | 2019.11-2020.11 | Changhong Town, Suzhuang Town, Hetian Town, Qixi Town | 267 | 140.39 |
| Total | | | | 1,271 | 2761.74 |

**Table S2.** Results of partial correlation analysis between damage types and factors in Zhejiang of eastern China.

| Damage types | Factors | *P* | Estimate |
|---|---|---|---|
| Total damages | vegetation area | 0.360 | 0.130 |
| | cultivated land | 0.322 | -0.140 |
| | GDP | 0.172 | -0.192 |
| | population gross | 0.117 | -0.220 |
| Number of damage to crops | vegetation area | 0.176 | 0.190 |
| | cultivated land | 0.271 | -0.155 |
| | GDP | 0.112 | -0.223 |
| | population gross | 0.229 | -0.170 |
| Area of damage to crops | vegetation area | 0.533 | 0.089 |
| | cultivated land | 0.683 | -0.058 |
| | GDP | 0.685 | -0.058 |
| | population gross | 0.748 | -0.046 |
| Amount of economic losses to residents | vegetation area | 0.577 | 0.079 |
| | cultivated land | 0.307 | -0.144 |
| | GDP | 0.475 | -0.101 |
| | population gross | 0.488 | -0.098 |

## Supplementary material Figures

Figure S1. Density of wild boar in each district and county we surveyed in Zhejiang of eastern China.
Figure S2. Changes of wild boar’s abundance in Zhejiang of eastern China.

**Figure S1.** Density of wild boar in each district and county we surveyed in Zhejiang of eastern China.

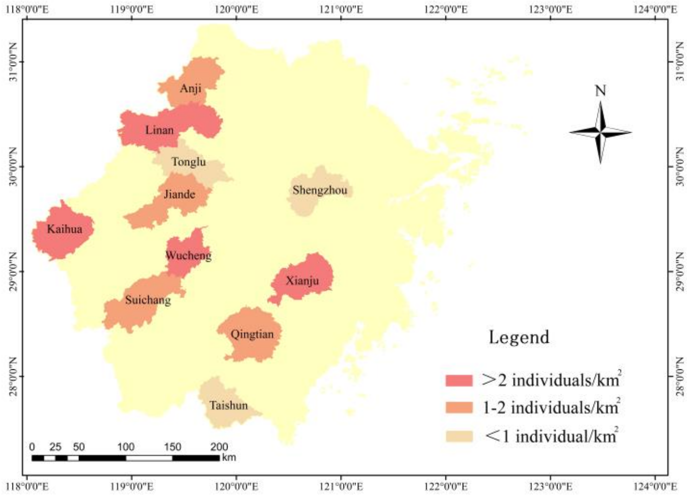


**Figure S2.** Changes of wild boar's abundance in Zhejiang of eastern China.

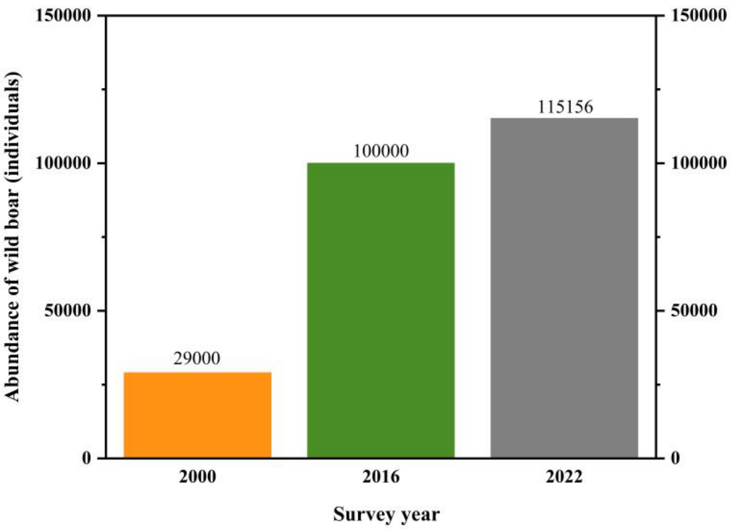

Supplement: Supplementary file 1 [file animals-14-01639-s001.zip › animals-3000089-supplementary.pdf]
